# Assessment of iron deposition in the heart in sickle cell patients using 3.0 Tesla cardiovascular magnetic resonance

**DOI:** 10.1186/1532-429X-14-S1-P194

**Published:** 2012-02-01

**Authors:** El-Sayed Ibrahim, Fauzia Rana, Kevin Johnson, Richard White

**Affiliations:** 1 Department of Radiology, University of Florida, Jacksonville, FL, USA; 2 Department of Medicine, University of Florida, Jacksonville, FL, USA

## Summary

This study investigates the role of 3.0T R2* imaging for measuring cardiac iron overload. Experiments were conducted on calibrated phantoms and sickle cell disease (SCD) patients. R2* imaging at 3.0T enabled assessment of wide range of iron concentrations. R2* values at 3.0T were about double of those at 1.5T. Myocardial R2* showed weak and strong correlations with hepatic R2* and serum ferritin, respectively. In conclusion, R2* imaging at 3.0T is promising technique for assessing cardiac iron overload in SCD with high sensitivity and reproducibility.

## Background

Heart failure due to cardiac iron overload is the leading cause of death in sickle cell disease (SCD) patients who receive frequent blood transfusion. CMR R2* imaging at 1.5T has been established for assessing iron overload. However, recent rapid growth in use of 3.0T scanners raises the question whether quantitative iron assessment will be possible at 3.0T, and what advantages are expected, which is investigated in this study.

## Methods

Ten gel-based phantoms were prepared with different iron concentrations (0-225µmol/g). The phantoms were imaged with optimized T2*-weighted sequence on Siemens 1.5T and 3.0T scanners (Fig.1). T2* was measured using monoexponential curve fitting (R2*=1000/T2*). The optimized 3.0T sequence was tested on 9 SCD patients. Imaging parameters: TR=200ms, matrix=256×192, FOV=380×285mm^2^, 10 echoes (TE=1.9-21.8ms), scan time=15s. Mid-ventricular short-axis heart slices at late diastole, and mid-liver transaxial slices were acquired (Fig.2). Average signal intensities were measured inside regions of the heart (septal wall) and liver. Regression analysis was conducted to study relationships between R2* at 1.5T, R2* at 3.0T, and iron concentration in the phantoms; and between myocardial R2*, liver R2*, and serum ferritin in patients. Bland-Altman analysis was conducted to measure inter- and intra-observer variabilities.

## Results

R2* values at 3.0T were about double of those at 1.5T. At 1.5T/3.0T, R2*(T2*) ranged from 50.7/112.4 1/s (19.7/8.9ms) to 158.7/344.8 1/s (6.3/2.9ms) at iron concentrations of 50 and 225µmol/g, respectively. T2* and R2* showed decreasing nonlinear and increasing linear (r=0.98/0.99) relationships, respectively, with iron concentration (Fig.1). At 1.5T, R2* of the lowest-concentration phantom (25 µmol/g) was almost zero. However, R2*(T2*) of this phantom was 34.8 1/s (28.7ms) at 3.0T, which was in agreement with the established linear relationship. In patients, myocardial/hepatic R2*(T2*) ranged from 32.6/78.7 1/s (30.7/12.7ms) to 78.1/243.9 1/s (12.8/4.1ms). The patients’ serum ferritin ranged from 50 to 3162ng/ml. Myocardial R2* showed weak (r=0.78) and strong (r=0.91) correlations with hepatic R2* and serum ferritin, respectively. Hepatic R2* had week correlation with serum ferritin (r=0.66). Bland-Altman showed no bias between repeated R2* measurements.

## Conclusions

R2* imaging at 3.0T enabled assessment of wide range of iron concentrations. However, it requires perfect shimming and very short echo times. The larger liver R2* compared to the heart indicates early iron deposition in liver. The good correlation between myocardial R2* and serum ferritin confirms that serum ferritin provides estimate of total body iron balance and correlates with R2* in absence of liver disease, inflammation, or infection. However, serum ferritin, by itself, is not reliable indicator of cardiac iron overload. In conclusion, R2* measurement at 3.0T is promising technique for assessing cardiac iron overload in SCD with high sensitivity and reproducibility.

## Funding

N/A

